# Fabrication and Characterizations of Pharmaceutical Emulgel Co-Loaded with Naproxen-Eugenol for Improved Analgesic and Anti-Inflammatory Effects

**DOI:** 10.3390/gels8100608

**Published:** 2022-09-22

**Authors:** Barkat Ali Khan, Sajeel Ahmad, Muhammad Khalid Khan, Khaled M. Hosny, Deena M. Bukhary, Haroon Iqbal, Samar S. Murshid, Abdulrahman A. Halwani, Mohammed Alissa, Farid Menaa

**Affiliations:** 1 Drug Delivery and Cosmetic Lab (DDCL), Gomal Centre of Pharmaceutical Sciences, Faculty of Pharmacy, Gomal University, Dera Ismail Khan 29050, Pakistan; 2 Department of Pharmaceutics, Faculty of Pharmacy, King Abdulaziz University, Jeddah 21589, Saudi Arabia; 3 Center of Excellence for Drug Research and Pharmaceutical Industries, King Abdulaziz University, Jeddah 21589, Saudi Arabia; 4 Department of Pharmaceutics, College of Pharmacy, Umm Al-Qura University, Makkah 24382, Saudi Arabia; 5 Institute of Basic Medicine and Cancer (IBMC), Chinese Academy of Sciences (CAS), Hangzhou 310022, China; 6 Department of Natural Products and Alternative Medicine, Faculty of Pharmacy, King Abdulaziz University, Jeddah 21589, Saudi Arabia; 7 Department of Medical Laboratory Sciences, College of Applied Medical Sciences, Prince Sattam Bin Abdulaziz University, Alkharj 11942, Saudi Arabia; 8 Departments of Internal Medicine, Pharmaceutics, and Nanomedicine, Fluorotronics Inc., Califormia Innovations Corp., San Diego, CA 92037, USA

**Keywords:** sustainability of natural resources, naproxen, eugenol, transdermal emulgel, skin permeation, inflammation, pain

## Abstract

The aim of this study was to fabricate and characterize a pharmaceutical emulgel co-loaded with naproxen/eugenol for transdermal delivery to improve the analgesic and anti-inflammatory effects and to eliminate GIT adverse reactions. Emulgel was prepared using a slow emulsification method and evaluated for physical appearance, thermodynamic stability, viscosity, pH, spreadability, extrudability, in-vitro drug release, drug content, ex-vivo permeation, drug retention studies and in-vivo studies. The emulgel exhibited good physical attributes, being thermodynamically stable with no phase separation, having excellent homogeneity, and pH 5.5 to 6.5. Slight changes in viscosity, spreadability and extrudability with respect to high temperature were observed (*p* > 0.05). The drug content was 96.69 ± 1.18% and 97.24 ± 1.27% for naproxen and eugenol, respectively. The maximum release of naproxen after 12 h was 85.14 ± 1.11%, whereas eugenol was 86.67 ± 1.23% from emulgel following anomalous non-Fickian mechanism. The maximum % permeation of naproxen across skin was 78.5 ± 1.30, whereas maximum % permeation of eugenol was 83.7 ± 1.33 after 12 h. The skin retention of eugenol and naproxen was 8.52 ± 0.22% and 6.98 ± 0.24%, respectively. The optimized emulgel inhibited the carrageenan induced paw edema. The pain reaction times of optimized emulgel and standard marketed product (Voltral^®^) were 11.16 ± 0.17 and 10.36 ± 0.47, respectively, with no statistically significant difference (*p* > 0.05). This study concluded that transdermal delivery of naproxen-eugenol emulgel synergized the anti-inflammatory and analgesic effects of naproxen and eugenol.

## 1. Introduction

Acute pain is an unacceptable and vigorous psychophysiological process, which may result in response to any injury in tissues or organs and involves many inflammatory progresses [1]. Acute pain is considered as physiological pain up to a certain limit; beyond that, it leads to the progression of chronic pain. There is no clear criterion for chronic pain; generally, it is recognized that pain that lasts for more than three months is chronic pain as per the international classification of disease [2]. Inflammation is a defensive biological response of body to harmful stimuli such as injured cells, chemicals, toxic compounds, and pathogens. It is the natural process to eliminate these harmful stimuli and promotes the healing process. Swelling, redness, pain, abnormal tissue function, and heat are some major symptoms of inflammation [3,4,5,6].

NSAIDs (non-steroidal anti-inflammatory drugs) are the class of most widely used drugs for their proved efficacy against pain and inflammation. NSAIDs constitute 5 to 10% of all medication prescribed every year [7,8,9]. Naproxen, an acid derivative of propionic acid, a non-selective COX inhibitor, is a well-known member of NSAIDs [5]. It used as an anti-inflammatory drug in the treatment of arthritis, gout, ankylosing spondylitis, and tendinitis [5,6]. Furthermore, naproxen also shows antipyretic and analgesic activity and is effective in the treatment of dysmenorrhea, rheumatoid arthritis, and post-operative pain [10,11,12].

Eugenol is an aromatic hydroxyl phenolic compound and obtained from *Cinnamomum zeylanicum, Ocimum basilicum, Myristica fragrans, Houtt gratissimum*, but its main source is *Eugenia caryophyllata* (Clove), which contains about 45 to 90% of eugenol [9,10]. Eugenol is included in the list of those essential oils which are considered as generally safe by the Food and Drug Administration [13]. Eugenol achieves its anti-inflammatory activity by competing with arachidonic acid to inhibit COX-2 enzymes, which in turn inhibits production of PGE2 (prostaglandin E2). It also inhibits the release of pro-inflammatory mediators like TNF (tumor necrosis factor-α) and interleukin 1 [14].

Drug products delivered through skin varies with respect to formulation ranging from liquid to powders in terms of consistency, but, among them, semisolid preparations gain more importance [15,16]. Topical semisolid preparations such as creams, ointments and lotions possess certain limitations such as stickiness and low spreadability causing low bioavailability and fluctuations in effective drug concentrations. Similarly, they also have a low amount of spreading coefficient and require rubbing when applied on the skin. Stability issues also limit their effectiveness. Owing to all these aspects, the transparent gel has improved the importance of transdermal routes both in cosmetics and in pharmaceutical preparations [17]. A transdermal drug delivery system serves as the best alternate to reduce and avoid the drawbacks associated with oral and parenteral delivery of drugs to the body [18].

In this research, presenting a unique work rather than an old idea has been tried. Naproxen, a non-steroidal anti-inflammatory drug, belongs to the Biopharmaceutics classification system (BCS) class II drug where the bioavailability is rate-limited by its dissolution i.e., having low aqueous solubility. In this study, the aforementioned research gap is attempted to be filled by formulating it as emulgel. Emulgel can load a high quantity of drugs with slow release and high solubility. Moreover, co-loading of drugs in a single topically applied transdermal dosage form can provide better compliance as taking two separate drugs is not easy and patients get exhausted. It avoids the GIT side effects related especially to naproxen. It will reduce costs because formulating two drugs separately required separate excipients, human resources, productions, and marketing costs. This study was conducted on an R&D basis, so it provides basic insights to conduct large scale commercial studies. Therefore, this study was designed to fabricate and characterize a pharmaceutical emulgel of naproxen and eugenol for analgesic and anti-inflammatory effects using an animal model.

## 2. Results and Discussion

### 2.1. Physical Appearance and Thermodynamic Stability

The stability of formulation is an important feature that determines the product consistency, therapeutic efficacy, and quality of drug product under different storage conditions of temperature, humidity, and pH [18]. The optimized naproxen–eugenol emulgel stored at different storage conditions for 28 days were observed for color, phase separation, smell, homogeneity, and grittiness. The results of physical stability parameters are represented in Table 1. It was observed that, during stability studies, physical parameters of optimized formulations at different storage conditions were stable; no phase separation, color change, and grittiness were seen in any storage condition up to 28 days. The stability of any product depends on the efficacy of surfactants, co-surfactants, co-solvents, preservatives, and environmental conditions. The surfactant (Tween-80) not only reduces the interfacial tension but is also committed to the stabilization of formulation [19]. The stabilization of optimized naproxen-eugenol emulgel might be due to the presence of Sepineo p600 (acrylamide/sodium acryloyldimethyl taurate copolymer). Bonacucina et al. reported the same findings; they investigated the emulsifying, self-gelling properties, and thickening agent of Sepineo P600 and concluded that Sepineo P600 can be a good polymer for stabilizing emulsion and gels with good rheological properties [20]. Similarly, the gelling properties of Carbopol have enhanced the stability of optimized emulgel, as Carbopol in the presence of water swells up at higher pH (above 4) and increases the thickness and stability of the formulation [21].

### 2.2. Evaluation of pH

The pH could be considered as an essential parameter for effective delivery, stability, and compatibility of topical formulations for human skin [18,19]. The pH of human skin ranges from 4.5 to 6.5, and it is believed that it must be within the range of physiological pH of skin; otherwise, it may cause irritation [20]. The results of pH evaluation for optimized emulgel at different temperatures (8 °C, 25 °C and 40 °C) and at time intervals (0, 1, 2, 7, 14, and 28 days) are displayed in Table 2. No significant variation in pH values were observed with respect to the duration at low temperature, while at 40 °C, significant decline was noted as compared to 8 °C and 25 °C. The initial readings at day 0 were 6.10 ± 0.04, which was decreased to 5.86 ± 0.38 after 28 days. Khan et al. elaborated that decline in pH of the formulation may be due to the oxidative property of liquid paraffin at high temperature, which upon oxidation liberates aldehydes and organic acids [21]. However, all the values of optimized formulation were in a normal range and acceptable for topical application. By applying a statistical tool, one way ANOVA, considering a 5% level of significance, the change in pH of optimized formulation at different temperatures was insignificant (*p* > 0.05).

### 2.3. Viscosity Determination

The viscosity plays a major role in the delivery of topically formulated drugs as it controls the release of drug at absorption site and thus ultimately influences the therapeutic efficiency of formulation [21]. The ingredients of any formulation such as polymers (gelling agent), surfactants, co-surfactants and oils greatly affect the viscosity of formulation [22]. The viscosity study of optimized formulations at different temperatures and time are presented in Table 3. The rheological properties of emulgel slightly decreased with increasing temperature and time. From day 0 to the 28th day, only 65 mPa/second declines were observed at 8 °C. At 25 °C, the decline is 447 mPa, whereas the 1607 mPa reduction was observed at 40 °C. Statistically, significant (*p* < 0.05) difference in viscosity with respect to temperature and duration of time was observed at 5% level of significance. The decrease in viscosity at higher temperature was also noted by Burki et al., and they proposed that the decrease in viscosity may be due to migration of water molecules from a dispersed oil phase to a continuous aqueous phase, and because of overflowing of numerous droplets due to osmotic pressure [14,23].

### 2.4. Spreadability Study

Spreadability is the property expressed to represent the extent of area to which formulation spread easily over the skin upon topical application [24]. Al-Suwayeh et al. have reported that therapeutic effectiveness of emulgel is affected by its spreadability value [25]. Similarly, Mishra et al. have reported that spreadability of emulgel is also an important factor for increasing patient compliance, as the formulation with high spreadability value provides more comfortable and uniform application over inflamed areas of skin [26]. The optimized formulation stored at different storage conditions, i.e., 8 °C, 25 °C, and 40 °C, were checked for the spreadability co-efficient. The readings were taken in triplicate and average ± sd were calculated. The result of spreading values of optimized formulation is presented in Table 4. Results show that spreadability value was low at 8 °C, i.e., 14.36 ± 0.92 as compared to 40 °C, which was 20.08 ± 1.43. This might be due to a decrease in viscosity at high temperature. The same phenomenon was described by Burki et al., claiming that spreadability of formulation is inversely proportional to the viscosity. By increasing viscosity, the spreading co-efficient decreases and vice versa [17]. The change in spreadability values of optimized naproxen-eugenol emulgel stored at different temperatures was significant (Student’s *t*-test *p* < 0.05).

### 2.5. Extrudability Study

Extrudability test is an important factor to be considered in the evaluation of semisolid dosage forms and is mainly dependent on the viscosity and consistency of formulation [19]. The extrudability values of emulgel stored at different temperature conditions have been presented in Table 5. The values observed were 20.03 ± 1.08, 19.89 ± 0.74, and 19.21 ± 0.53 at temperatures 8 °C, 25 °C and 40 °C, respectively. High extrusion value at low temperature and low extrudability reading at elevated temperature indicates the direct relation between viscosity and extrusion rate [26]. Adegbenro and Opeyemi suggested that low viscous formulations exhibit better extrudability, which in turns improves patient compliance [27]. By using a statistical tool, the Student *t*-test considering a 5% level of significance (*p* < 0.05), the change in extrudability values of optimized formulation at different temperatures was significant (*p* < 0.05).

### 2.6. Drug Content Assays

The drug content uniformity is an essential parameter which directly affects the therapeutic efficacy of any pharmaceutical dosage form and can be determined by the percentage assay of drug or active pharmaceutical ingredient [17]. The percent drug content of naproxen and eugenol from emulgel was 96.69 ± 1.18% and 97.24 ± 1.27%, respectively, shown in Table 6. The results of percent drug contents indicated that the drug was uniformly distributed, and the result was within the limit (90–110%) according to US Pharmacopeia. Moreover, no drug loss was observed during manufacturing and processing of optimization of formulation.

### 2.7. In-Vitro Drug Release Study

**Figure 1:**
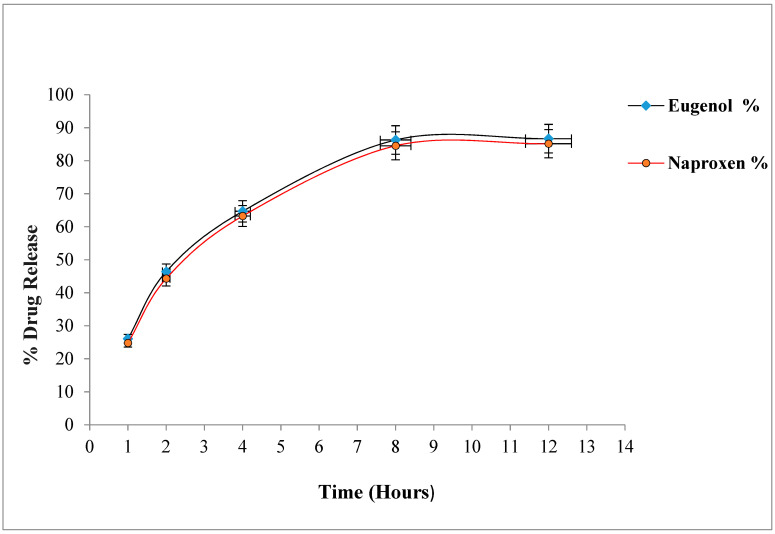
Percentage drug release of naproxen and eugenol from emulgel.

The therapeutic efficacy of any drug depends upon the drug release from pharmaceutical dosage forms [7]. The percentage release of naproxen and eugenol from optimized emulgel is shown in Figure 1. The percentage release of eugenol was 86.67 ± 1.23%, whereas naproxen showed a percentage release of 85.14 ± 1.11% after 12 h. The drug release pattern from the topical or transdermal dosage form is influenced by various factors such as polymers, gelling agents, concentration of surfactants, spreading co-efficient, and rheological properties [28]. The concentration of gelling agent greatly affects the release of drug from dosage form i.e., an increase in concentration of polymers decreases the diffusion through the membrane [29]. However, this did not happen with our optimized emulgel, possibly because of the presence of co-solvents. Song et al. reported that ethanol and propylene glycol increase the solubility of drugs, and propylene enhances the uptake of drug into the skin, whereas ethanol decreases the viscosity of Carbopol [30].

#### Drug Release Kinetics

The results obtained from the release kinetics of naproxen and eugenol by applying different drug release kinetics models were summarized in Table 7. The interpretation of the best fitted release model was based on the highest value of regression co-efficient (R^2^) closer to (1) [19]. From the results, it is observed that R^2^ value of naproxen (R^2^ = 0.994) and eugenol (R^2^ = 0.992) are the highest in the Korsmeyer–Peppas model among all applicable models, so they obeyed the Korsmeyer–Peppas model. The value of *N* is greater than 0.5 for both drugs, i.e., Naproxen (*N* = 0.784) and Eugenol (*N* = 0.719), which indicates that the drug followed the anomalous non-Fickian, zero order release mechanism [10].

### 2.8. Ex-Vivo Permeation Study

**Figure 2:**
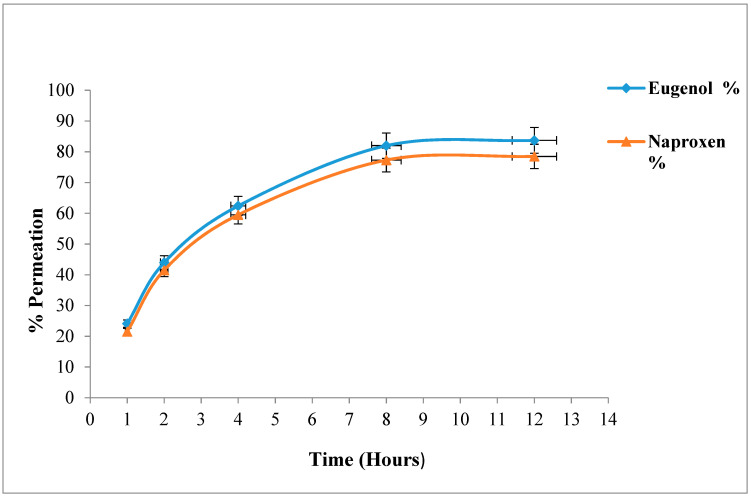
Cumulative permeation of naproxen and eugenol from optimized emulgel.

Drug permeation across skin is the main contributing factor for efficient delivery of drug via a transdermal route [30]. The percentage permeations of naproxen and eugenol across rabbit skin from emulgel are presented in Figure 2. The maximum permeation of naproxen across skin was 78.5 ± 1.30, whereas maximum % permeation of eugenol was 83.7 ± 1.33 after 12 h. Various factors like concentration of surfactants, co-solvents, penetration enhancers and skin contact time are involved in drug permeation across skin [17]. When surfactants meet the skin, they bind with skin proteins, causing protein denaturation and leading to the swelling of the stratum corneum. Tween-80 increases the skin permeability by the leakage of low molecular mass compounds from the phospholipid membrane [31]. Co-solvents, ethanol and propylene glycol increase the solubility of drugs. Similarly, ethanol also decreases the viscosity of Carbopol [22]. The higher skin permeation of eugenol as compared to naproxen might be because eugenol itself acts as a drug permeation enhancer. The same finding was reported by Pramod et al.—that penetration enhancement of clove oil was due to eugenol. Eugenol was evaluated for in-vitro (procine epidermis) and in-vivo (on rabbit and mouse skin) penetration enhancement activity for transdermal drug delivery systems, and good penetrating results were obtained every time [32].

### 2.9. Drug Retention

The drug retention in the skin can be considered as an important parameter for the treatment of local skin disorders [33]. The percentage drug retention of naproxen and eugenol from optimized emulgel are given in Table 8. The skin retention of eugenol and naproxen was 8.52 ± 0.22 and 6.98 ± 0.24, respectively. The higher drug retention of eugenol in rabbit skin might be due to the skin penetration properties of eugenol [18]. The results are in the accordance studies that evaluate the antioxidant and skin retention properties of eugenol gel and its derivative (eugenyl dichloroacetate) and reported that greater skin retention of eugenol was observed as compared to its derivative from gel base [33,34].

### 2.10. In-Vivo Characterization of Naproxen-Eugenol Emulgel

#### 2.10.1. Skin Irritation Study

In transdermal drug delivery systems, the active ingredients are sometimes associated with skin irritation if retained for a longer duration of time after application [18,19]. Thus, it is essential to evaluate the interaction between formulation and skin regarding skin sensitivity. The signs (erythema and edema) for skin irritation of the control group (placebo) and test group (naproxen-eugenol emulgel) observed for three days are presented in Table 9. It was observed that, after application of emulgel, the skin color of one rat in the test group was observed to be red, which disappeared after two days. However, in the placebo group, no edema or erythema were observed. The slight morphological change of skin color in the test group might be due to the presence of eugenol because of its skin irritating effects as reported earlier [35].

#### 2.10.2. In-Vivo Analgesic Activity

**Figure 3:**
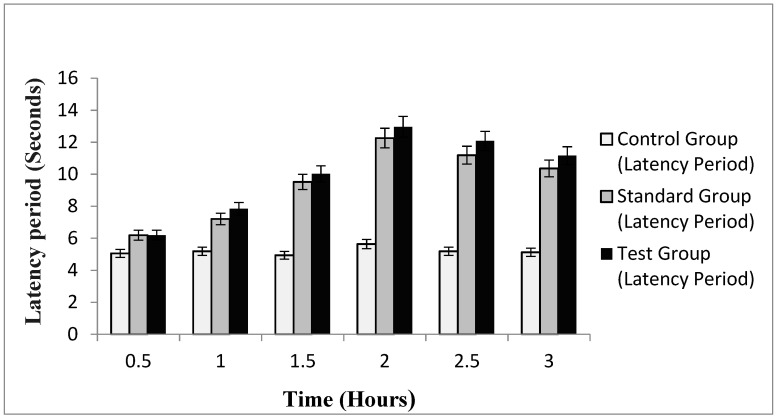
Latency period of control, standard and test group.

The efficacy of emulgel in enhancing transdermal therapeutic effects of naproxen and eugenol from emulgel was evaluated using a hot plate method for determining in-vivo analgesic activity. The hot plate method is considered a central anti-nociceptive test for in-vivo studies [35]. The mean pain reaction times of control group (without treatment), standard group (treated with Voltral^®^ emulgel, GSK, Karachi, Pakistan) and test group (treated with optimized naproxen-eugenol emulgel) at different time intervals are summarized in Figure 3. The results indicated that, after 30 min, the mean pain reaction time of standard group and test group was 6.19 ± 0.19 and 6.22 ± 0.32, respectively, and it was observed that up to 2 h latency time continued to increase in both groups. The maximum response noted by the standard group was 12.26 ± 0.12, whereas the test group showed a maximum reaction time of 12.96 ± 0.9. The higher analgesic activity of optimized emulgel may be due to the synergistic effect of naproxen and eugenol. Eugenol exhibits skin permeating effects along with analgesic activity. The same findings were reported by Taher et al.; they evaluated the anti-nociceptive and anti-inflammatory activity of clove oil in mice and claimed that analgesic activity of clove oil is due to eugenol, and it had greatly increased the pain reaction time in mice upon exposure to a hot plate [36]. The pain reaction time of optimized emulgel was significantly enhanced (*p* < 0.05) as compared to the control group, whereas the difference in standard and test group was insignificant (*p* > 0.05).

#### 2.10.3. In-Vivo Anti-Inflammatory Activity

**Figure 4:**
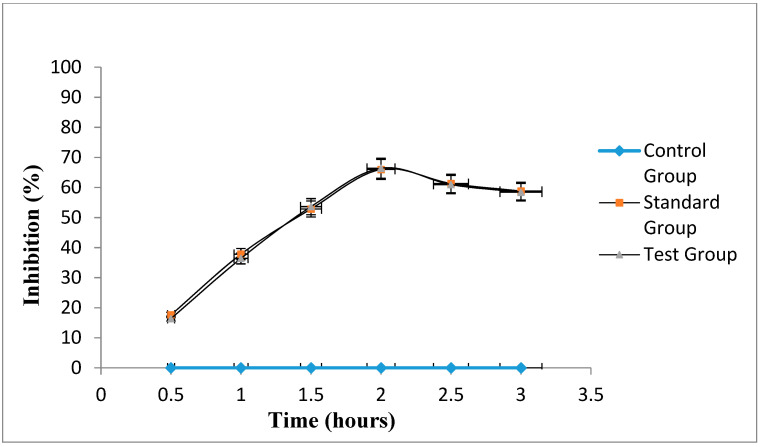
Percent edema inhibition of the standard group and test group.

For the assessment of in-vivo anti-inflammatory activity of experimental drugs, carrageenan induced edema could be considered a standard experimental model, which shows highly predictive results [37]. Figure 4 represents the inhibition of edema induced by carrageenan in the standard group and test group against control group at different indicated times. In the beginning, the test group exhibited approximately similar inhibitions to that of the standard group, but after 1.5 h, the inhibitory activity of the test group was slightly increased (4.11 ± 0.16) compared to the standard group, which was (4.12 ± 0.10) and maximum activity was noted at 2 h. After 2 h, the percentage inhibition of marketed formulation (Voltral^®^) and optimized naproxen-eugenol emulgel continued to decrease. The same phenomenon was observed by the Cong et al. when they evaluated the therapeutic intensity and duration of NSAIDs by assessing anti-inflammatory activities of naproxen and indomethacin; after 5 h, no anti-inflammatory activity was observed, and they concluded that NSAIDs inhibit the early signs of inflammation more effectively, and it is well known that NSAIDs had limited outcomes in chronic conditions [38,39]. Statistically, the emulgel had significantly (*p* < 0.05) inhibited and reduced edema in the hind paws of rats. However, the difference in percentage inhibition of standard group and test group was insignificant (*p* > 0.05). The dual (penetration enhancement and anti-inflammatory) properties of eugenol had not only improved the permeation of naproxen across skin but also synergized the in-vivo anti-inflammatory effects of optimized emulgel. Eventually, transdermal permeation of drugs stabilized in rationally designed and optimally characterized drug carriers, such as naproxen-eugenol emulgel, is a critical parameter to consider enhancing the effects of drugs (e.g., pain and inflammation) while minimizing their side-effects [40,41,42].

## 3. Conclusions

From the obtained results, it can be concluded that optimized emulgel formulation with Carbopol and sepineo-p600 possessed desired thermodynamic and other physicochemical stability. The release pattern of the drug from optimized formulation followed the Korsmeyer–Peppas model. The present study has affirmed that the developed optimized formulation is a promising alternative for better pain management.

## 4. Materials and Methods

### 4.1. Chemicals

All chemicals used were of analytical grade which include Naproxen Sodium (Divi’s Laboratories Limited, Hyderabad, India), Eugenol and Tween-80 (Sigma-Aldrich Chimie S.a.r.l, Saint-Quentin-Fallavier, France), Propylene glycol (Dongying HI-Tech Spring Chemicals, Shandong, China), Ethanol (BDH laboratory supplies, Poole, UK), Liquid paraffin (Al-Noor petroleum, Sindh, Pakistan), Carbopol 934 (Sigma-Aldrich, Taufkirchen, Germany), Sepineo-P600 (Alternative Chemica (Pvt.) Ltd., Punjab, Pakistan), Methanol (Chem-Lab Analytical bvba, Zedelgem, Belgium), Carrageenan (Sigma-Aldrich, St. Louis, MO, USA) and Voltral^®^ (GSK, Karachi, Pakistan).

### 4.2. Apparatus

A digital pH meter (Milwaukee Instruments, Inc., Rocky Mount, NC, USA), Weighing balance (Sartorius, Tokyo, Japan), Magnetic stirrer (78HW-1, ZENITH LAB(JIANGSU) CO., LTD, Jintan, China), Water bath 1042 (GFL Gesellschaft für Labortechnik mbH, Burgwedel, UK), Viscometer NDJ-8S, (Drawell International Technology Ltd., Chongking, China), Sonicator Bath (Marten Walter Ultraschalltechnik AG, Straubenhardt, Germany), Franz diffusion cell (JN SCIENCETECH, Hyderabad, India), Vernier caliper (CD-6′′ ASX, TOTAL Calibration Solutions Inc., Mentor, OH, USA), Refrigerator PEL PRLP-2350 (PAKREF, Karachi, Pakistan), Stability chamber WS-150 (Guangdong Jinuosh Technology Co., Ltd., Dongguan City, China), UV-visible spectrophotometer Shimadzu-1700 (Shimadzu, Tokyo, Japan) and HPLC Shimadzu-SPD-20AT (Shimadzu, Tokyo, Japan) were used in the experiments.

### 4.3. Preparation of Naproxen-Eugenol Emulsion

The naproxen-eugenol emulsion was prepared by the method adapted by Burki et al. with minor modifications [14]. Aqueous phase was prepared by adding tween 80 to distilled water and stirring for 3–5 min. Then, weighed quantities of methylparaben sodium and propylparaben sodium were dissolved in propylene glycol, and the mixture was incorporated into a solution of water and tween 80. Naproxen was dissolved in ethanol and added to the aqueous phase with constant stirring. After 5–10 min, sepineo-P600 was added to the aqueous phase and allowed the aqueous phase to stir at moderate speed. Oil phase was prepared by adding eugenol to olive oil. Both phases were separately heated up to 70 °C, and oil phase was added to the aqueous phase dropwise and stirred at 1500 rpm for 50 to 60 min. The composition of optimized formulation is given in Table 10.

### 4.4. Preparation of Gel

Accurately weighed 2 g of Carbopol 934 was dispersed in 100 mL distilled water with constant stirring at moderate speed and kept overnight to form a thick gel network. pH was adjusted to 5–6 by adding a few drops of triethanolamine.

### 4.5. Preparation of Naproxen-Eugenol Emulgel (NEE)

The emulsion was incorporated into a gel base in a 1:1 ratio until a viscous gel network of NEE was formed and pH was adjusted.

### 4.6. Physical Appearance

Six different batches (Pre-formulation data not shown) of naproxen-eugenol emulgel were incubated at 25 °C for 28 days and evaluated for color change, homogeneity, creaming/phase separation and grittiness. Based on results of physical parameters, the optimized formulation was selected and further subjected to in-vitro, in-vivo and ex-vivo studies.

### 4.7. Determination of pH

The pH of optimized naproxen-eugenol emulgel formulation was determined by a digital pH meter which was stored at different temperatures (8 °C, 25 °C and 40 °C), and, at the intervals of 0, 1, 2, 7, 14 and 28 days, the pH values were noted. The test was performed in triplicate, and average values were calculated [15].

### 4.8. Rheological Study

The viscosity of optimized formulations was determined according to a slightly modified method previously published [15]. The viscometer NDJ-8S (Korea) with spindle no. 4 was used for viscosity determination at temperature 8 °C, 25 °C and 40 °C at the intervals of 0, 1, 2, 7, 14 and 28 days. The spindle was dropped vertically into the center of beaker containing optimized emulgel in such a way that it did not touch the bottom of the beaker, the spindle was rotated at 6 rpm for five minutes, and readings were observed. The tests were performed in triplicate, and average values were calculated.

### 4.9. Determination of Drug Assays

The chemical assay of naproxen and eugenol in emulgel was determined by using a U/V spectrophotometer and HPLC respectively, as reported in literature [16]. It has been explained below in detail.

#### 4.9.1. Preparation of Standard Naproxen Solution

The standard stock solution of naproxen was prepared by taking 25 mg of naproxen in a 25 mL volumetric flask. It was dissolved in 10 to 15 mL of methanol and sonicated for 2–3 min. The remaining volume was made with methanol up to the mark. A required concentration, i.e., 30 µg/mL, was prepared by transferring 1.5 mL from stock solution into a 50 mL volumetric flask, and the final volume was made with methanol up to the mark. The standard solution was then filtered through 0.45-micron filter paper.

#### 4.9.2. Preparation of Sample Naproxen Solution

The sample stock solution of naproxen was prepared by taking 500 mg of optimized naproxen/eugenol emulgel equivalent to 25 mg of naproxen in a 25 mL volumetric flask. It was dissolved in 10 to 15 mL of methanol and sonicated for 10 min. The remaining volume was made with methanol up to the mark, filtered through a 0.45-micron filter. The required concentration, i.e., 30 µg/mL, was prepared by transferring 1.5 mL of this solution into a 50 mL volumetric flask, the final volume was made with methanol and maximum absorbance was noted at λ_max_ 331 nm:

(1) $$\%\;\mathrm{Assay}=\frac{\mathrm{Absorption}\;\mathrm{of}\;\mathrm{sample}}{\mathrm{Absorption}\;\mathrm{of}\;\mathrm{standard}}\times 100$$

#### 4.9.3. Preparation of Mobile Phase for Eugenol

The mobile phase was prepared by mixing methanol and distilled water in a ratio of 60:40, which was then passed through a filter having a pore size of 0.45 microns (Nylon membrane, Sigma-Aldrich, St. Louis, MO, USA). After filtration, the mobile phase was degassed by placing it in the sonicator for 10 min.

#### 4.9.4. Preparation of Eugenol Standard Solution

The standard stock solution of eugenol was prepared by taking 25 mg of eugenol in a 25 mL volumetric flask. It was dissolved in 10 to 15 mL of methanol and sonicated for 2–3 min. The remaining volume was made with methanol up to the mark. The required concentration, i.e., 40 µg/mL, was prepared by transferring 1 mL from stock solution into a 25 mL volumetric flask, and the final volume was made with methanol up to the mark. The standard solution was filtered through 0.45-micron filter paper.

#### 4.9.5. Preparation of Eugenol Test Solution

The sample stock solution of eugenol was prepared by taking 1 g of optimized naproxen/eugenol emulgel equivalent to 10 mg of eugenol in a 50 mL volumetric flask. It was dissolved in 20 to 25 mL of methanol and sonicated for 10 min. The remaining volume was made with methanol up to the mark, filtered through a 0.45-micron filter. The required concentration, i.e., 40 µg/mL, was prepared by transferring 5 mL of this solution into a 50 mL volumetric flask, and final volume was made with methanol up to the mark.

#### 4.9.6. Procedure

A C-18 column (5 μm, C18, 150 × 4.6 mm id) was used for this procedure. Mobile phase consisted of methanol and water in a 60:40 ratio, and the flow rate was 0.8 mL/min (Isocratic flow). The injection volume was 20 μL, and the effluent was observed at 280 nm. The temperature was set at 30 °C and retention time was 5 min. A specified volume (20 µL) of standard and sample solutions was separately injected into the chromatograph. The chromatograms were recorded, and the responses of the individual components peaks in standard and sample eugenol solution were measured. The results were taken in triplicate, and average values were calculated. The percent assay of eugenol was determined by using the following formula:

(2) $$\%\;\mathrm{Assay}=\frac{\mathrm{Peak}\;\mathrm{response}\;\left(\mathrm{area}\right)\;\mathrm{of}\;\mathrm{sample}}{\mathrm{Peak}\;\mathrm{response}\;\left(\mathrm{area}\right)\mathrm{of}\;\mathrm{standard}}\times 100$$

### 4.10. Spreadability Study

Spreadability of emulgel was determined by a drag and slip apparatus according to the method adapted by Akram et al. [18]. The apparatus consists of a wooden block to which a pulley was attached on its one edge and a glass slide was fitted over the wooden block. Approximately 2 g of emulgel were taken over the glass slide attached to the wooden block and sandwiched by placing another glass slide having the same dimensions as that of the fixed glass slide. About 80 g weight was suspended by fastening a string to the upper glass slide with the help of a hook and passed above the pulley; the time in seconds by the slide to cover 7.5 cm on the fixed glass slide was noted. The spreading co-efficient was then calculated by using the following formula:

Spreadability = (M × L)/T(3)

where

M = weight tied to upper glass slide,
L = length of glass slides,
T = time taken to separate the glass slides from each other.

### 4.11. Extrudability Study

The extrudability test is performed to measure the force that is required to extrude the emulgel from a collapsible tube. The evaluation of formulation extrudability was based upon the quantity of emulgel extruded from aluminum collapsible tubes by applying weight in grams required to extrude at least a 0.5-cm ribbon of emulgel from tube in 10 s. This test was performed in triplicate, and average values were calculated [15].

The extrudability of emulgel was then calculated by using the following formula:

Extrudability = ((Weight in gram) to extrude emulgel)/(Area (cm^2^)) (4)

### 4.12. In-Vitro Drug Release

The in-vitro drug release studies of optimized formulation were carried out using a Franz diffusion cell. The cellulose acetate membrane (Sartorius, Tokyo, Japan) having a pore size of 0.45 µm was clamped between the donor and receptor compartment. Prior to the loading of formulation, the receptor compartment was filled with a 5 mL phosphate buffer pH 5.5, and temperature was maintained at 37 ± 1 °C under constant stirring at 300 rpm. About 1 gram of optimized emulgel equivalent to (naproxen = 100 mg and eugenol = 20 mg) was placed on the donor compartment. The samples were drawn at specific time intervals, i.e., at time 0, 1, 2, 4, 8 and 12 h, and were replaced with 2 mL fresh phosphate buffer to maintain the sink condition. All the samples were then analyzed by a UV visible spectrophotometer at 282 and 331 nm for eugenol and naproxen, respectively.

### 4.13. Drug Release Kinetics

The data obtained from in-vitro drug release studies were evaluated for kinetics of drug release from the naproxen-eugenol emulgel by employing the different mathematical kinetic models such as zero order kinetic, first order kinetic, the Korsmeyer–Peppas model, the Hixson–Crowell model, the Higuchi model, and the model having a high correlation co-efficient was considered the best fitting model.

### 4.14. Ethical Consideration

For ex-vivo studies, permission for animal use was granted from the institutional ethical review board (ERB) of Gomal University Dera Ismail Khan, Pakistan under reference No. 851/QEC/GU. All the protocols were followed according to the NIH guidelines for lab animals.

### 4.15. Ex-Vivo Permeation Study

The ex-vivo permeation study was carried out using a Franz diffusion cell. The excised rat skin, previously refrigerated, was first hydrated with normal saline at least 1.5 h before experiment and then clamped between the donor and receptor compartment of the Franz diffusion cell in such a way that the epidermis layer of skin interacts with the donor compartment. Prior to the loading of formulation, the receptor compartment was filled with phosphate buffer pH 7.4. About 1 g of emulgel was placed on the donor compartment, and a 2 mL sample was drawn at specific time intervals, i.e., at time 0, 1, 2, 4, 8 and 12 h, and was replaced with 2 mL of fresh phosphate buffer to maintain the sink condition. All the samples were then analyzed by a UV visible spectrophotometer at 282 and 331 nm for eugenol and naproxen, respectively.

### 4.16. Skin Retention Study

The skin was removed from a Franz diffusion cell and washed with phosphate buffer (7.4) to remove the drug on the surface of skin. The skin was then cut into small pieces and dropped into the beaker containing methanol to extract the drugs from skin and stirred for 2 h. The solution was filtered, and samples were analyzed by a UV visible spectrophotometer at 282 and 331 nm for eugenol and naproxen retention, respectively.

### 4.17. Skin Irritation Study

For the skin irritation study, rats were divided into control and test groups containing 4 rats in each group (*n* = 4). Control group was treated with blank emulgel, and the test group was treated with naproxen-eugenol emulgel. The blank emulgel and drugs loaded emulgel were applied over the hairless rat’s skin at intervals of 8 h. After 24 h, the skin was washed with water, the signs of irritation (erythema and edema) were observed for the period of 0, 1, 2 and 3 days, and sensitivity reactions were scored as 0 (no reaction), 1 (slightly erythema), 2 (moderate erythema) and 3 (severe erythema with or without edema). The total score for skin irritation was calculated using the following equation:

Primary irritation index = (Erythema reaction score + edema reaction score)/(Time Interval (h))(5)

### 4.18. In-Vivo Anti-Inflammatory Activity

The in-vivo anti-inflammatory activity of optimized emulgel was carried out according to the method adapted by Khan et al. and slightly modified [17]. For this purpose, 0.1 mL of 1% carrageenan injection was administered in the sub planter part of rats for inducing edema in their right hind paws. Before half an hour of carrageenan injection, both standard formulation and test formulation were applied topically to the right hind paw of rats. The volume of edema was measured by using a Vernier caliper immediately after carrageenan injection at time zero and then at intervals of 0.5, 1, 1.5, 2, 2.5 and 3 h. The percentage inhibition of the test group was measured and compared with the control group and standard group by using the following equation:

% Inhibition = (Vc − Vt)/Vt × 100(6)

where

Vc = inflammatory increase in paw volume of the control group,
Vt = inflammatory increase in paw volume of the test group.

#### Treatment Protocols

For in-vivo anti-inflammatory activity, animals were divided into 3 groups containing 6 rats in each group (*n* = 6) as follows:

- **Group 1**: Control group, Treated with 0.1 mL of 1% carrageenan injection only,
- **Group 2:** Test group, Treated with optimized naproxen-eugenol emulgel + 0.1 mL of 1% carrageenan injection.
- **Group 3**: Standard group, Treated with marketed emulgel (Voltral^®^) + 0.1 mL of 1% carrageenan injection.

### 4.19. In-Vivo Analgesic Activity

The in-vivo analgesic activity of optimized emulgel formulation was determined by a hot plate method [17]. The temperature of the hot plate was maintained at 55 ± 1 °C, and each rat from all three groups, i.e., control group, test group and standard group, were placed over the hot plate and pain reaction time or latency time was noted using stopwatch after topical application of standard and test formulations at time intervals of 0.5, 1, 2, 2.5 and 3 h. The response to pain stimuli (jumping, licking, and rising of hind feet) of the test group was calculated and compared with the standard and control groups.

For in-vivo analgesic activity, animals were divided into 3 groups containing 6 rats in each group (*n* = 6) as follows:

- **Group 1**: Control group, no treatment was given,
- **Group 2**: Test group, Treated with optimized naproxen-eugenol emulgel,
- **Group 3**: Standard group, Treated with marketed diclofenac emulgel (Voltral^®^).

## 5. Statistical Analysis

All the experiments were performed in triplicate, and the results were expressed as average ± sd. The statistical analysis was carried out by using ANOVA and the Student’s *t*-test considering *p* < 0.05 as statistically significant.

## Figures and Tables

**Table 1:** Physical parameters of naproxen-eugenol emulgel in different storage conditions.

Stability Parameters		Day 0	Day 1	Day 2	Day 7	Day 14	Day 28
**Color**	**A**	LY	LY	LY	LY	LY	LY
	**B**	LY	LY	LY	LY	LY	LY
	**C**	LY	LY	LY	LY	LY	LY
	**D**	LY	LY	LY	LY	LY	LY
**Homogeneity**	**A**	***	***	***	***	***	***
	**B**	***	***	***	***	***	***
	**C**	***	***	***	***	**	**
	**D**	***	***	***	**	**	**
**Smell**	**A**	-ve	-ve	-ve	-ve	-ve	-ve
	**B**	-ve	-ve	-ve	-ve	-ve	-ve
	**C**	-ve	-ve	-ve	-ve	-ve	-ve
	**D**	-ve	-ve	-ve	-ve	-ve	-ve
**Phase separation**	**A**	-ve	-ve	-ve	-ve	-ve	-ve
	**B**	-ve	-ve	-ve	-ve	-ve	-ve
	**C**	-ve	-ve	-ve	-ve	-ve	-ve
	**D**	-ve	-ve	-ve	-ve	-ve	-ve
**Grittiness**	**A**	-ve	-ve	-ve	-ve	-ve	-ve
	**B**	-ve	-ve	-ve	-ve	-ve	-ve
	**C**	-ve	-ve	-ve	-ve	-ve	-ve
	**D**	-ve	-ve	-ve	-ve	-ve	-ve

LY = light yellow, -ve = No smell, ** Good rating for homogeneity, *** Excellent rating for homogeneity, -ve = no phase separation, -ve = No grittiness, A = at 8 °C (60%), B = at 25 °C (60%), C = 30 °C (65%), D = 40 °C (75%).

**Table 2:** pH of optimized naproxen-eugenol emulgel at different temperatures and duration of time.

Time (Days)	pH at 8 °C	pH at 25 °C	pH at 40 °C
0	6.10 ± 0.04	6.10 ± 0.04	6.10 ± 0.04
1	6.08 ± 0.07	6.09 ± 0.40	6.06 ± 0.94
2	6.07 ± 0.01	6.07 ± 0.23	6.02 ± 0.36
7	6.03 ± 0.28	6.04 ± 0.34	5.94 ± 0.68
14	6.03 ± 0.97	6.02 ± 0.45	5.88 ± 0.24
28	6.01 ± 0.18	5.98 ± 0.12	5.86 ± 0.38

All the values are calculated as mean ± sd, (*n* = 3).

**Table 3:** Viscosities of optimized naproxen-eugenol emulgel at different temperatures and time.

Time (Days)	Viscosity (mPa) at 8 °C	Viscosity (mPa) at 25 °C	Viscosity (mPa) at 40 °C
0	19,687 ± 0.17	19,687 ± 0.17	19,687 ± 0.17
1	19676 ± 0.18	19,594 ± 0.40	19,260 ± 0.94
2	19,650 ± 0.18	19,390 ± 0.91	19,120 ± 0.32
7	19,638 ± 0.12	19,318 ± 0.34	18,680 ± 0.68
14	19,630 ± 0.23	19,290 ± 0.45	18,320 ± 0.28
28	19,622 ± 0.23	19,240 ± 0.12	18,080 ± 0.40

All the values are calculated as mean ± sd, (*n* = 3).

**Table 4:** Spreading co-efficient of optimized emulgel formulation at different temperatures.

S.No	Temperature	Spreadability (gm·cm/s)
1	8 °C	14.36 ± 0.92
2	25 °C	16.12 ± 1.36
3	40 °C	20.08 ± 1.43

All the values are calculated as mean ± sd, (*n* = 3).

**Table 5:** Extrudability of optimized emulgel formulation at different temperatures.

S.No	Temperatures	Extrudability (gm/cm^2^)
1	8 °C	20.03± 1.08
2	25 °C	19.89 ± 0.74
3	40 °C	19.21 ± 0.53

All the values are calculated as mean ± sd, (*n* = 3).

**Table 6:** Drug content of naproxen and eugenol from emulgel.

Drugs	Standard (Mean ± sd)	Sample (Mean ± sd)	Drug Content (Mean ± sd)
Naproxen (Absorbance)	0.242 ± 0.006	0.234 ± 0.01	96.69%
Eugenol (Area under the peak)	1,036,891 ± 1.26	1,008,324 ± 1.79	97.24%

**Table 7:** Drug release kinetics of optimized emulgel.

Drugs	Zero Order Kinetic		1st Order Kinetic		Higuchi	Hixson–Crowell			Korsmeyer–Peppas		
	K	R^2^	K	R^2^	K	R^2^	K	R^2^	K	R^2^	*N*
**Eugenol**	1.29	0.98	1.46	0.59	1.14	0.99	1.0	0.991	1.14	0.99	0.71
**Naproxen**	1.86	0.98	1.65	0.65	1.14	0.99	1.2	0.993	1.01	0.99	0.78

**Table 8:** Skin retention of naproxen and eugenol from optimized emulgel.

S.No	Drugs	% Drug Retention
1	Naproxen	6.98 ± 0.24
2	Eugenol	8.52 ± 0.22

All the values are calculated as mean ± sd (*n* = 3).

**Table 9:** Skin irritation scoring of control group and test group at different time intervals.

Days	Primary Irritation Index	
	Control Group	Test Group
0	0.00	0.00
1	0.00	0.04
2	0.00	0.02
3	0.00	0.00

**Table 10:** Composition of optimized naproxen-eugenol emulsion formulations (100 g, *w*/*w*).

S.No	Ingredients	Quantity (*w*/*w*)
1	Naproxen	10
2	Eugenol	2
3	Tween-80	10
4	PG	8
5	Olive oil	6
6	Ethanol	4
7	Liquid paraffin	6
8	Methyl paraben	0.03
9	Propyl paraben	0.05
10	Sepineo-P600	6
12	Distilled water q.s	100

## Data Availability

Not applicable.
